# A phase I/II study of oxaliplatin when added to 5-fluorouracil and leucovorin and pelvic radiation in locally advanced rectal cancer: a Colorectal Clinical Oncology Group (CCOG) study

**DOI:** 10.1038/sj.bjc.6602818

**Published:** 2005-10-25

**Authors:** D Sebag-Montefiore, R Glynne-Jones, S Falk, H M Meadows, T Maughan

**Affiliations:** 1 Cookridge Hospital, Hospital Lane, Cookridge, West Yorkshire LS16 6QB, UK; 2 Mount Vernon Hospital, Rickmansworth Road, Northwood, Middlesex HA6 2RN, UK; 3 Bristol Oncology Centre, Bristol Royal Infirmary, Horefield Road, Bristol BS2 8ED, UK; 4 Cancer Research UK & UCL Cancer Trials Centre, Stephenson House, 158-160 North Gower Street, London NW1 2ND, UK; 5 Velindre Hospital, Whitchurch, Cardiff CF4 7XL, UK

**Keywords:** 5-fluorouracil, oxaliplatin, locally advanced rectal cancer, preoperative chemoradiation

## Abstract

The purpose of this study was to evaluate the maximum tolerated dose (MTD) and recommended dose of oxaliplatin given synchronously with 5-fluorouracil (5FU), leucovorin (LV) and preoperative pelvic radiation for primary unresectable, locally advanced, rectal cancer. Preoperative pelvic radiotherapy using a three- or four-field technique and megavoltage photons comprised 45 Gy given in 25 fractions, 1.8 Gy per fraction, and delivered with escalating doses of oxaliplatin in combination with low-dose LV and 5FU. Chemotherapy was given synchronously with radiotherapy in weeks 1 and 5. Escalating doses of oxaliplatin (85, 130 and 150 mg m^−2^) were given on days 2 and 30, followed by low-dose LV (20 mg m^−2^) and 5FU (350 mg m^−2^), both given on days 1–5 and 29–33. Surgery was performed 6–10 weeks later. The MTD was determined as the dose causing more than a third of patients to have a dose-limiting toxicity (DLT). Once the MTD was reached, a further 14 patients were treated at the dose level below the MTD. In all, 32 patients received oxaliplatin at the three dose levels, median age 60 years (range 31–79), 24 males and eight females. The MTD was reached at 150 mg m^−2^ when four out of six patients experienced DLT. Dose-limiting grade 3 or 4 diarrhoea was reported in two out of six patients at 85 mg m^−2^, 5 out of 20 at 130 mg m^−2^ and four out of 6 at 150 mg m^−2^. Grade 3 neuropathy was reported at 130 mg m^−2^ (1 out of 20) and at 150 mg m^−2^ (two out of six), and serious haematological toxicity was minimal; one grade 3 anaemia at 150 mg m^−2^. In all, 28 out of 32 patients completed all treatments as planned; three had radiotherapy interrupted and three a chemotherapy dose reduction. Four patients did not proceed to surgery due to the presence of metastatic disease (two), unfitness (one) or patient refusal (one). Also, 28 patients underwent surgical resection. Histopathology demonstrated histopathological complete response (pCR) 2 out of 27 (7%), Tmic 3 out of 27 (11%), pCR+Tmic 5 out of 27 (19%), pT0–2 6 out of 27 (22%) and histologically confirmed clear circumferential resection margins in 22 out of 27 (81%). Dose-limiting toxicity with oxaliplatin is 150 mg m^−2^ given days 2 and 30 when added to the described 5FU LV and 45 Gy radiation preoperatively. The acceptable toxicity and compliance at 130 mg m^−2^ recommend testing this dose in future phase II studies. The tumour downstaging and complete resection rates are encouragingly high for this very locally advanced group.

A curative surgical resection remains the most important component of the modern multimodality management of rectal cancer. However, randomised controlled trials (Swedish Rectal Cancer Trial, 1997; Kapiteijn *et al*, 2001; Sauer *et al*, 2004) and two meta-analyses (Cammà *et al*, 2000; Colorectal Cancer Collaborative Group, 2001) have also demonstrated that adjuvant radiation can reduce the rate of locoregional recurrence, although its impact on overall survival is less clear. The meta-analyses show greater benefit for preoperative radiotherapy treatment.

Recent advances that have improved the outcome in rectal cancer include a more radical surgical technique incorporating mesorectal excision (Enker *et al*, 1995, Heald *et al*, 1998) and a more accurate histopathological examination of the resected specimen, reporting the proximity of microscopic tumour to the circumferential resection margin (CRM) (Quirke *et al*, 1986). Individual surgical series, a population-based audit (Wibe *et al*, 2002) and evidence from a randomised controlled trial (Nagtegaal *et al*, 2002), all demonstrate lower rates of local recurrence following mesorectal excision compared with the previous randomised trials that included a surgery alone arm. In addition, involvement of the CRM is associated with a significantly increased risk of local recurrence and also metastatic disease.

There is an increasing use of preoperative chemoradiation (CRT) in patients with locally advanced rectal cancer, despite the fact that there are few trials that have compared preoperative CRT with radiation alone (Frykholm *et al*, 2001; Bosset *et al*, 2004). The aims of treatment are to reduce the extent of the primary tumour to allow macroscopic removal to take place, treat potential microscopic disease close to or beyond the mesorectal fascia and, if present, also treat micrometastatic disease outside the radiation fields.

Most CRT schedules in current use combine pelvic radiation with either continuous infusion 5-fluorouracil (5FU) or 5FU modulated by leucovorin (LV). Mature results from the EORTC 22921 and FFCD 9203 trials of operable T3/T4 rectal cancer have recently demonstrated a convincing reduction in locoregional failure when a CRT regimen of 5FU and LV (as used in this study) is added to 45 Gy of pelvic radiation (Bosset *et al*, 2004, 2005; Gerard *et al*, 2005). Phase III trials in metastatic colorectal cancer have demonstrated statistically significant improvements in the overall response rate and progression-free survival when oxaliplatin is added to a combination of 5FU LV (de Gramont *et al*, 2000; Grothey, 2002; Goldberg *et al*, 2004). In the adjuvant setting, the addition of oxaliplatin to 5FU LV significantly improves disease-free survival (DFS) (André *et al*, 2004). As the EORTC 22921 and FFCD 9203 trials used 5FU LV-based CRT schedules, it was a logical step to develop preoperative radiation schedules that incorporate the addition of oxaliplatin to this regimen. The choice of the 5FU LV regimen required the oxaliplatin to be given in weeks 1 and 5 to ensure concomitant administration.

Oxaliplatin is also recognised to have radiosensitising effects in both cell cultures and mouse xenografts. In preclinical models of combined radiotherapy and oxaliplatin, an 8-h oxaliplatin exposure was associated with a dose-related cell kill rate (Blackstock *et al*, 2002). Synergistic effects with radiation were observed when oxaliplatin was given both before and after radiation. In mouse xenograft models of colorectal cancer, tumour growth has been shown to be inhibited by combined oxaliplatin and radiation (Blackstock *et al*, 2000).

The present study aimed to evaluate the addition of two doses of oxaliplatin 4 weeks apart to a regimen of CRT that uses folinic acid and 5FU daily during the first and fifth weeks of pelvic radiation (45 Gy), as used in the EORTC 22921 and FFCD 9203 phase III trials (Bosset *et al*, 2004; Gerard *et al*, 2005).

## PATIENTS AND METHODS

### Study design

**Figure 1:**
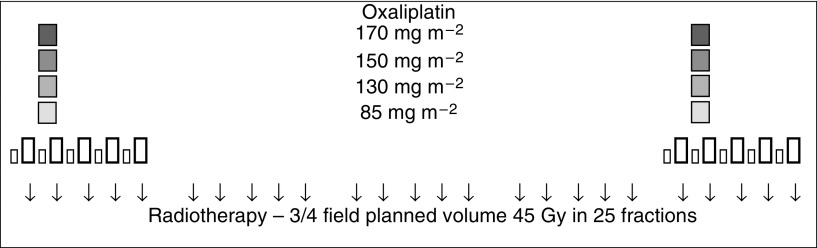
Chemoradiation schedule. □ Oxaliplatin at dose levels shown on days 2 and 30 prior to LV, 5FU; 
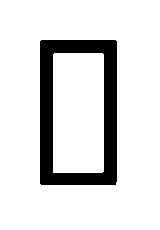
, LV 20 mg m^−2^ bolus on days 1–5 and 29–33; ↓, 5FU 350 mg m^−2^ 60-min infusion on days 1–5 and 29–33; ↓, radiotherapy 1.8 Gy per fraction.

This dose escalation study aimed to increase the dose of oxaliplatin given on days 2 and 30 in successive cohorts when added to a CRT regimen consisting of low-dose LV and a short infusion of 5FU administered concurrently with radiation until the maximum tumour dose was reached. All patients received radiotherapy (45 Gy) with LV (20 mg m^−2^) and 5FU (350 mg m^−2^ administered over 1 h) on days 1–5 and 29–33 (see Figure 1). There were four planned oxaliplatin dose levels, each consisting of six patients. Oxaliplatin was administered in 250 ml dextrose over 2 h on days 2 and 30 (85, 130, 150, 170 mg m^−2^). Dose-limiting toxicities (DLTs) were defined to include grade III or IV diarrhoea, nausea, vomiting, thrombocytopaenia and neutropaenia according to the National Cancer Institute Canada common toxicity criteria revised 1994 definitions and grade III acute sensorimotor neuropathy (i.e. functional impairment). Toxicity was recorded prospectively weekly up to and including week 10.

If grade III or IV DLT was observed in two or less patients in a cohort of six, then a further cohort was treated at the next dose level. The maximum tolerated dose (MTD) was defined if three or more of six patients experienced DLT. Once the MTD was defined, the preceding dose level would then be expanded and a total of 20 patients treated at the recommended dose.

The primary end points of the study were acute grade III/IV toxicity and compliance with the planned doses of chemotherapy and radiotherapy. Secondary end points included the histopathological complete response (pCR) rate, resectability rate, local recurrence rate and late morbidity.

### Eligibility criteria

Eligibility criteria included histologically confirmed adenocarcinoma, WHO performance status 0–2 and no evidence of metastatic disease using chest X-ray and abdominopelvic CT. Acceptable haematological and renal function was required: neutrophils >1.5 × 10^9^ l^−1^, platelets ⩾100 × 10^9^ l^−1^, and serum creatinine <1.25 × the institutions upper limit of normal range.

Patients with locally advanced, biopsy-proven carcinoma of the rectum were included either on the basis of fixity on digital rectal examination (DRE), T4 stage on pelvic CT or when MRI demonstrated a high risk of involvement of the CRM. The method used to define locally advanced disease, the distance of the inferior tumour border to the anal verge (cm) and intended surgical procedure were also recorded.

Patients were excluded from the study because of prior chemotherapy or pelvic radiation, lack of efficient contraception or pregnancy, inflammatory bowel disease and cardiac conditions that would deter the safe delivery of 5FU. Patients having six episodes of stool per day or who were incontinent of faeces were also excluded.

### Pelvic radiation

Using information from clinical examination and pelvic MRI, the gross tumour volume (GTV) was defined using a CT planning scan. To derive the planning target volume (PTV), margins were added to the GTV according to the radiation planning diagrams (3 cm laterally, superiorly and inferiorly, 2 cm anteriorly) included in the protocol, with the exception of the posterior border, which was always located on the most posterior aspect of the bony sacrum. Patients were treated prone with a full bladder using either a three- or four-field technique. A total dose of 45 Gy was delivered to the International Commission on Radiation Units intersection point using 25 daily fractions of 1.8 Gy. Orthogonal film simulation was performed with opacification of the small bowel using barium sulphate 300 ml with gastrograffin 20 ml.

### Surgery and histopathology

Surgery was recommended to take place 6–10 weeks after completion of CRT. Histopathological examination of the resected specimen was performed according to the technique described by Quirke (Birbeck and Quirke, 1999). The CRM is considered involved if microscopic tumour is present at or less than 1 mm from the circumferential or radial resection margin.

### Assessment during and after treatment

Full blood count, urea creatinine and electrolytes, and liver function tests and acute toxicity scores were assessed prospectively on weeks 1–6 and 10. On completion of CRT, follow-up appointments were given at 3, 6 12, 24 and 36 months to assess tumour recurrence and late toxicity.

For the purposes of this study, local recurrence has been defined in patients who have had a complete macroscopic resection as evidence of either an intraluminal or extraluminal mass below the sacral promontory and biopsy-proven adenocarcinoma, or CEA abnormal with or without the presence of metastases. Radiological evidence of interval enlargement of the mass (minimum interval of 6 weeks) was required if based on CT scans alone (biopsy negative and CEA normal).

## RESULTS

Between February 1999 and October 2001, a total of 32 patients were recruited from four centres: 24 males, eight females, median age 60 years (range 31–79). Their tumour characteristics are summarised in Table 1. Five patients were excluded from the prospective assessment of DLT and dose recommendations. Two patients failed to start treatment, two patients refused the day 30 dose of oxaliplatin, but had not experienced DLT, and one patient died during CRT of an intestinal perforation considered unrelated to study therapy.

### Protocol compliance

During the dose escalation phase, full compliance was achieved (no reduction in planned dose or duration of treatment) for radiation dose or chemotherapy dose at 85 mg m^−2^, whereas one out of six patients at 130 mg m^−2^ required interruption of radiation and three out of six patients at 150 mg m^−2^ required chemotherapy dose reduction (two of which also had radiotherapy interrupted).

The median field size, in all patients, for the posterior/anterior field was 15.1 cm (range 10.5–20.1) height × 13.9 cm (range 11.4–18.0) width, and for the lateral/oblique fields 15.4 cm (range 11.5–20.0) height × 13.4 cm (range 10.5–20.5) width. In all, 28 out of 32 patients proceeded to have radical surgery; the median time to surgery was 9 weeks (interquartile range 8–11 weeks). Information on pathological status was available on 27 patients due to the loss of one surgical specimen.

### Acute toxicity

The acute grade III/IV toxicity experienced is summarised for the three dose levels and the subsequent patients treated in the expanded cohort at 130 mg m^−2^ in Table 2. During the dose escalation phase, DLT was seen in two out of six patients at 85 mg m^−2^ and in four out of six patients at 150 mg m^−2^. In view of the toxicity seen at 150 mg m^−2^, the decision was made to stop recruitment to this dose level and to expand the 130 mg m^−2^ dose level to the planned total of 20 patients.

The most common toxicity was diarrhoea; this occurred in 10 patients, eight grade 3 and two grade 4. Three patients had grade 3 neurotoxicity, 15 others reported this toxicity at grade 1 or 2, all of these events resolved within 4weeks following treatment. Haematological toxicity was rare, with only one grade 3 anaemia reported at the 150 mg m^−2^ dose level.

### Response to treatment

Tumour downstaging was defined by comparing clinical TN stage prior to treatment (as determined by pelvic MRI) with histopathological stage post surgery. Chemoradiation achieved downstaging in 8 out of 27 (30%) of patients. The pathological stages at surgery were T0N0=2, T1NO=1, T0N1=1, T1N1=1, T2N0=3, T3N0=9, T3N1=6, T3NX=1 and T4N0=3. It can be seen that CRT achieved a complete pathological response in 2 out of 27 (7%) cases who proceeded to surgery (Table 3) and 2 out of 32 (6%) of the whole group. Histopathology also demonstrated microscopic disease only (Tmic) in 3 out of 27 (11%), patients, pCR+Tmic in 5 out of 27 (19%), pT0–2 in 6 out of 27 (22%) and histologically confirmed (>1 mm) clear CRM in 22 out of 27 (81%).

### Surgery

**Figure 2:**
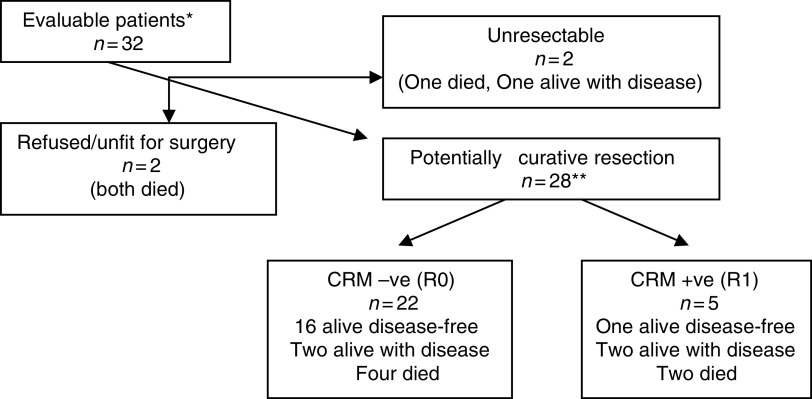
Patient outcome. ^*^Five unassessable patients not included; one died, two did not start treatment, two refused week 5 chemo; ^**^one specimen lost.

Of the 32 evaluable patients, four did not proceed to surgery due to the presence of metastatic disease (two), patient refusal (one) and unfitness for surgery (one) (Figure 2). All patients had a preliminary clinical assessment by their surgeon at entry, and were categorised on the basis of height of the cancer from the anal verge as requiring an abdominoperineal resection (APR) or anterior resection (AR). Of the 28 surgical procedures, 15 had an APR, two of which had initially been assessed as requiring an AR, the remaining 13 had APR as expected (one with cystectomy). Ten patients had an AR, six of which had been anticipated and four that had been expected to require an APR. One patient had a Hartman's procedure after an initial assessment of an APR. Two additional patients required exenterative surgery (one initially assessed to require this procedure and one initially assessed as requiring AR). There were no unexpected post operative complications or deaths.

### Local recurrence and survival

**Figure 3:**
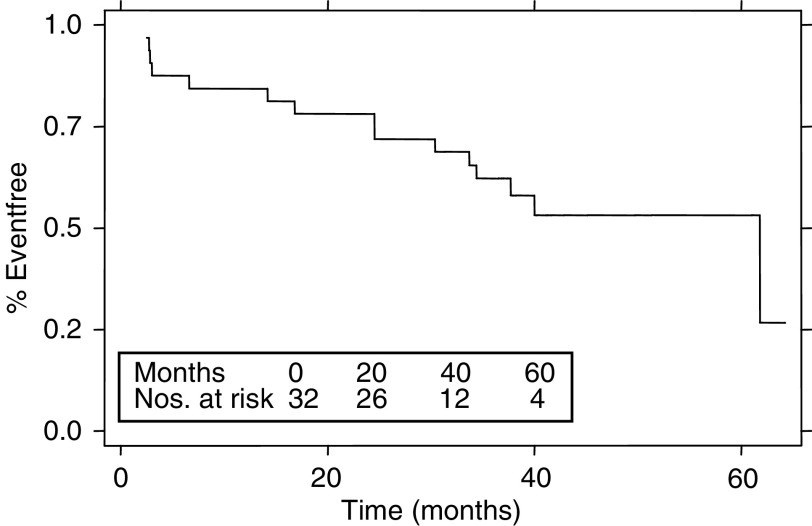
Disease-free survival – all patients.

The strength of this study is that the median follow-up is 41 months (range 6–64 months) for all patients and 41 months for surviving patients (range 31–64 months). In all, 23 of the 32 patients remain alive at the time of writing. Of the 22 patients having complete surgical clearance of tumour (CRM−ve) six relapsed (one local, four distant, and one at local and distant sites), of which four subsequently died of disease (Figure 2 and Table 4). Of the five incomplete resections (CRM+ve) only one patient is alive without disease (45 months from the start of treatment); the remaining four progressed: one local, one distant, and two local and distant, both of whom died. Three of the four patients who were not resected died and the fourth patient is alive at 45 months with a pelvic mass. The DFS for the whole group at 3 years is 62% (95% CI 43–77%) (see Figure 3) and 82% (95% CI 58–93%) for those having had a complete resection. All six patients who had T0–2 NO tumours remain alive and disease free. Of the 21 patients with T3/4 NO/1 or T0–2 N1 disease, 11 are alive and disease free, four are alive with disease and six have died due to disease.

### Late effects

Severe late effects were uncommon, despite the combination of oxaliplatin and radiotherapy. Grade 3 late toxicity was reported in five patients (85 mg m^−2^; one diarrhoea, 130 mg m^−2^; one tenesmus and diarrhoea, 150 mg m^−2^; one tenesmus and one bleeding and pain). As four of the five patients had relapsed disease, it is unclear whether the symptoms reflect radiation or recurrence.

## DISCUSSION

The objective of this study was to determine the MTD of oxaliplatin in combination with pelvic radiotherapy and low-dose folinic acid and a 60-min infusion of 5FU with 45 Gy of radiotherapy given preoperatively in patients with locally advanced rectal cancer. The eligibility criteria used in this study define a relatively homogeneous group of very locally advanced rectal cancer. The MTD of oxaliplatin combined with pelvic radiotherapy (using relatively large field size) has been defined as 150 mg m^−2^ on days 2 and 30, and the recommended dose as 130 mg m^−2^. The dose intensity of oxaliplatin (260 mg m^−2^ over 5 weeks) in the present study is similar to that achieved by Freyer *et al* (2001) using a day 1 and 29 schedule and higher than that used (200 mg m^−2^) in a recent German study (Rödel *et al*, 2003).

The histologically confirmed RO (CRM –ve) resection rate of 81% is high for this locally advanced group (Bosset *et al*, 2005; Gerard *et al*, 2005). In this study, only two patients had unresectable disease and only three patients had partial pelvic clearances. In contrast, the German CAO/ARO/AIO – 94 study (Rödel *et al*, 2003) reported 8 out of 31 patients who underwent exenteration or resection of adjacent organs.

This study did not seek to assess the impact or control the use of postoperative chemotherapy. Only 7% of the study group received such treatment and this consisted of 5FU LV. The EORTC 22921 trial (Bosset *et al*, 2005) did not demonstrate a significant impact on outcome when postoperative chemotherapy was compared with no further treatment when given after preoperative CRT, and it is unlikely that the use of postoperative chemotherapy will have significantly altered the outcome of this study.

Enthusiasm for preoperative CRT in the management of rectal cancer is increasing. The German CAO/ARO/AIO – 94 study protocol has convincingly shown improved locoregional control and reductions in acute and late toxicity with preoperative CRT (Sauer *et al*, 2004) *vs* postoperative combined modality treatment for stage II/III resectable rectal cancer. The rationale for preoperative CRT is attractive, as it combines early systemic chemotherapy treatment simultaneously with a locoregional treatment.

In addition, over the past decade, nonrandomised studies have confirmed that preoperative treatment with radiotherapy (Wagman *et al*, 1998) can facilitate sphincter-sparing options. A study from Lyon compared immediate and delayed surgery after preoperative radiotherapy (Francois *et al*, 1999). Sphincter preservation was achieved in 79% of patients with a long interval (4–6 weeks) following radiotherapy, compared with 69% of patients where the interval was only short (2 weeks). A further report (Glehen *et al*, 2003) documents that, with a median follow-up of 6.3 years, no differences were found in the degree of local control, overall survival, morbidity, anal function or surgical complications between the two groups. The addition of chemotherapy to radiotherapy can be used to shrink the primary tumour further and facilitate sphincter-sparing options. Impressive results appear to have been achieved in phase II studies with CRT (Grann *et al*, 1997; Rullier *et al*, 2001) and long-term follow-up has confirmed an excellent outcome if there is marked shrinkage of the primary tumour (Ruo *et al*, 2002).

There have been several reported phase I and II studies integrating oxaliplatin into fluoropyrimidine-based CRT schedules (Gérard *et al*, 2003; Gambacorta *et al*, 2004; Aschele *et al*, 2005). There are, however, important differences between these and the current study. Gambacorta *et al* (2004) evaluated the combination of raltitrexed and oxaliplatin with 50.4 Gy radiation. Aschele *et al* (2005) used a weekly schedule of oxaliplatin combined with continuous infusion 5FU and 50.4 Gy irradiation. Gérard *et al* (2003) used the same doses of 5FU/LV as this study, but delivered this as a continuous infusion over 24 h for days 1–5 and 29–33, but did not determine the MTD of the combination of oxaliplatin on days 1 and 29 when added to 5FU/LV and radiation.

The present study has a number of important differences from the above studies. Firstly, the total dose of radiation was fixed at 45 Gy, a total dose that is 10% lower than the other studies. This dose might be expected to be associated with a lower incidence of late complications. Secondly, this study formally determined the MTD and recommended dose of oxaliplatin when added to a validated CRT fluoropyrimidine schedule used in two recent phase III trials (Bosset *et al*, 2005; Gerard *et al*, 2005). Finally, this study reports outcome data that is relatively mature (41-month median follow-up) and is also based on the circumferential margin status. We are not aware that any other combination CRT study has reported such data. This is superior to retrospective data with 5FU CRT (Sebag-Montefiore *et al*, 2005). Now that there is clear evidence that locoregional control is improved by the addition of the 5FU/LV regimen used in this study to 45 Gy of pelvic radiation, there is a strong rationale for future trials to establish the benefit of the addition of oxaliplatin.

Further studies by our group, including the CORE study (DSM personal communication) abstract submitted to ECCO and Socrates studies (Gerard *et al*, 2005; Glynne-Jones *et al*, 2003, 2005), will further clarify the potential of using oxaliplatin in combination with oral fluoropyrimidines and radiotherapy in locally advanced rectal cancer. These data suggest that combination radiochemotherapy leads to improved early histopathological outcome measures. This has the potential to translate into improved long-term outcomes in rectal cancer, both in terms of quality of life and overall survival, and will be tested in current and future phase III trials.

In conclusion, the MTD with oxaliplatin is confirmed as 150 mg m^−2^ given on days 2 and 30, when added to the described 5FU LV and 45 Gy radiation preoperatively. The recommended dose of oxaliplatin in this setting is 130 mg m^−2^. This dose is associated with high compliance and manageable acute toxicity, and appears to offer significant shrinkage and pathological downstaging even for bulky T3/T4 cancers considered unresectable. In the adjuvant setting, the addition of oxaliplatin to 5FU/LV significantly improves DFS (André *et al*, 2004) and may reduce the incidence of micrometastases. There are no concerns regarding excess late morbidity from this combination, and we therefore recommend testing this dose in future phase II/III studies.

## Figures and Tables

**Table 1:** Patient and tumour characteristics

**Characteristic**	***n*=32**
Median age (range)	60 (31–79)
Male : female	24 : 8
WHO status 0 : 1 : 2	9 : 21 : 2
	
*Site of tumour*	
Upper : mid : lower	4 : 12 : 16
	
*Local extent*	
Fixed/unresectable	16
Locally advanced on MRI	16

WHO=World Health Organisation; MRI=magnetic resonance imaging.

**Table 2:** Acute toxicity

**Oxaliplatin dose (mg m^−2^)**	**85**	**130^a^**	**150**	**All**
No. of patients	6	6/14	6	32
Patients with DLT	2	2/3	4	11
Gd 3 diarrhoea	2	0/3	3	8
Gd 4 diarrhoea	0	2/0	0	2
Gd 3 neurological^b^	0	1/0	2	3
Gd 3 anaemia	0	0/0	1	1

a The figures in this column indicate the numbers in the initial six patients treated at this dose level, followed by the numbers in the expanded group once the MDT was determined.

b Defined as functional impairment using the Sanofi oxaliplatin neurotoxoicity score.

**Table 3:** Pathological response

**Oxaliplatin dose (mg m^−2^)**	**85**	**130**	**150**	**All (%)**
Number of patients	6	20	6	32
Number operated	6	19^a^	3^b^	28
pCR	0/6	2/19	0/2	2/27 (7%)
pT0–2 pN0	2/6	6/19	0/2	8/27 (30%)
Tmic	1/6	2/19	0/2	3/27 (11%)
−ve CRM^c^	5/6	15/19	2/2	22/27 (81%)

a One patient inoperable.

b One inoperable, one unfit for surgery, one patient refused, one specimen lost.

c −ve CRM=tumour clearance of more than 1 mm from circumferential resection margin, pCR=histopathological complete response; Tmic=microscopic disease only detected in surgical specimen; CRM=circumferential resection margin.

**Table 4:** Pattern of recurrence following radical resection

**Oxaliplatin dose (mg m^−2^)**	**85**	**130**	**150**	**All**
Number of patients	6	19	3	28
Any disease	3	7	1	11/27 (41%)
Local recurrence	1	3	0	4/27 (15%)
Distant metastases	3	5	1	9/27 (33%)
Death	3	3	1	7/27 (26%)
